# 2-*p*-Tolyl-2,3-di­hydro­quinolin-4(1*H*)-one

**DOI:** 10.1107/S1600536814001676

**Published:** 2014-01-29

**Authors:** Meryem Chelghoum, Abdelmalek Bouraiou, Sofiane Bouacida, Mebarek Bahnous, Ali Belfaitah

**Affiliations:** a Laboratoire des Produits Naturels d’Origine Végétale, et de Synthèse Organique, PHYSYNOR Université Constantine 1, 25000 Constantine, Algeria; b Unité de Recherche de Cimie de l’Environnement et Moléculaire Structurale, CHEMS, Université Constantine 1, 25000 , Algeria; c Département Sciences de la Matière, Faculté des Sciences Exactes et Sciences de la Nature et de la Vie, Université Oum El Bouaghi, 04000 Oum El Bouaghi, Algeria

## Abstract

In the title mol­ecule, C_16_H_15_NO, the tetra­hydro­pyridine ring is in a sofa conformation with the methine C atom forming the flap. The dihedral angle between the benzene rings is 80.85 (8)°. In the crystal, mol­ecules are arranged in alternating double layers parallel to (100) and are connected along [001] by N—H⋯O hydrogen bonds. In addition, weak C—H⋯π inter­actions are observed.

## Related literature   

For applications of quinolines, see: Hepworth (1984 ▶). For the synthesis and applications of similar compounds see: Donnelly & Farrell (1990*a* ▶,*b* ▶); Chandrasekhar *et al.* (2007 ▶); Kumar *et al.* (2004 ▶); Gordon (2001 ▶); Olivier-Bourbigou & Magna (2002 ▶); Tokes & Szilagyi (1987 ▶); Tokes & Litkei (1993 ▶); Benzerka *et al.* (2012 ▶, 2013 ▶); Hayour *et al.* (2011 ▶) Chelghoum *et al.* (2012 ▶). For related structures, see: Tokes *et al.* (1992 ▶); Benzerka *et al.* (2011 ▶); Bouraiou *et al.* (2011 ▶).
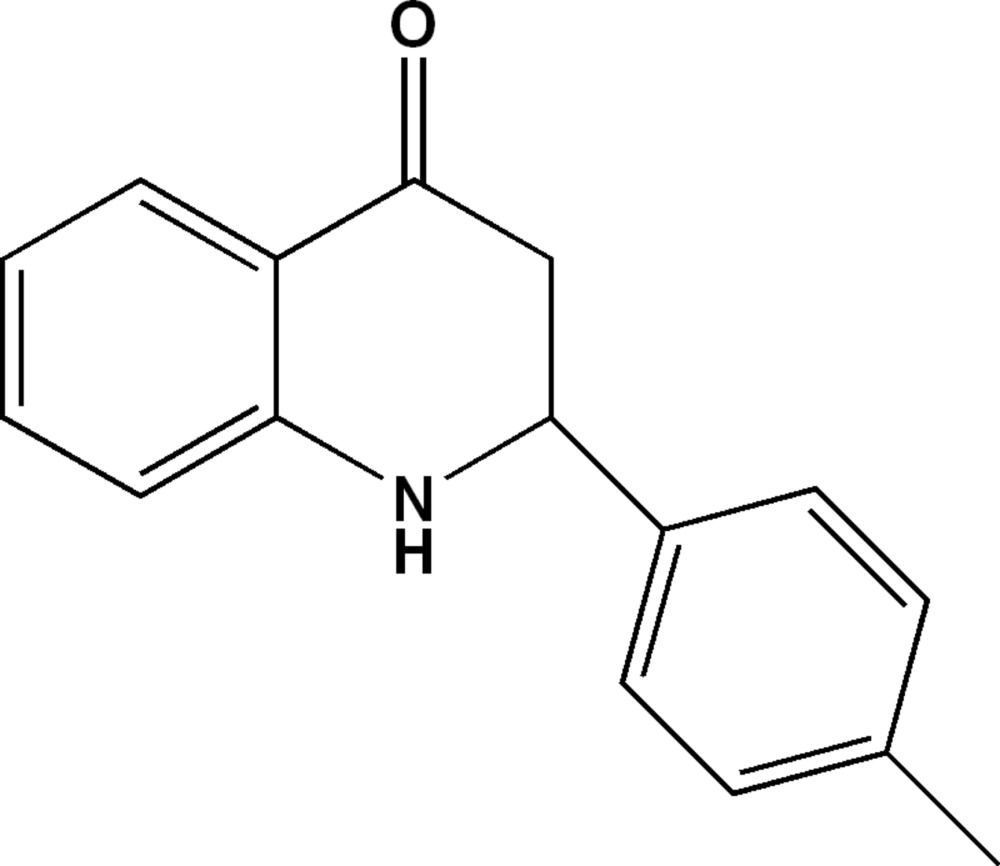


## Experimental   

### 

#### Crystal data  

C_16_H_15_NO
*M* *_r_* = 237.29
Monoclinic, 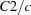

*a* = 17.6363 (14) Å
*b* = 10.7968 (9) Å
*c* = 13.6308 (9) Å
β = 103.260 (3)°
*V* = 2526.3 (3) Å^3^
*Z* = 8
Mo *K*α radiation
μ = 0.08 mm^−1^
*T* = 150 K
0.52 × 0.33 × 0.27 mm

#### Data collection  

Bruker APEXII diffractometer
Absorption correction: multi-scan (*SADABS*; Sheldrick, 2002 ▶) *T*_min_ = 0.873, *T*_max_ = 0.979
6746 measured reflections
2875 independent reflections
2279 reflections with *I* > 2σ(*I*)
*R*_int_ = 0.026

#### Refinement  

*R*[*F* ^2^ > 2σ(*F* ^2^)] = 0.053
*wR*(*F* ^2^) = 0.153
*S* = 1.05
2875 reflections
167 parameters
H atoms treated by a mixture of independent and constrained refinement
Δρ_max_ = 0.69 e Å^−3^
Δρ_min_ = −0.28 e Å^−3^

### 

Data collection: *APEX2* (Bruker, 2006 ▶); cell refinement: *SAINT* (Bruker, 2006 ▶); data reduction: *SAINT*; program(s) used to solve structure: *SIR2002* (Burla *et al.*, 2005 ▶); program(s) used to refine structure: *SHELXL97* (Sheldrick, 2008 ▶); molecular graphics: *ORTEP-3 for Windows* (Farrugia, 2012 ▶) and *DIAMOND* (Brandenburg & Berndt, 2001 ▶); software used to prepare material for publication: *WinGX* (Farrugia, 2012 ▶) and *CRYSCAL* (T. Roisnel, local program).

## Supplementary Material

Crystal structure: contains datablock(s) I. DOI: 10.1107/S1600536814001676/lh5684sup1.cif

Structure factors: contains datablock(s) I. DOI: 10.1107/S1600536814001676/lh5684Isup2.hkl

Supporting information file. DOI: 10.1107/S1600536814001676/lh5684Isup3.cml

CCDC reference:

Additional supporting information:  crystallographic information; 3D view; checkCIF report

## Figures and Tables

**Table 1:** Hydrogen-bond geometry (Å, °) *Cg*1 and *Cg*2 are the centroids of the C13–C18 and C3–C9 rings, respectively.

*D*—H⋯*A*	*D*—H	H⋯*A*	*D*⋯*A*	*D*—H⋯*A*
N1—H1*N*⋯O12^i^	0.88 (2)	2.09 (2)	2.9484 (17)	166.9 (18)
C4—H4⋯*Cg*1^ii^	0.95	2.70	3.546 (2)	149
C14—H14⋯*Cg*2^iii^	0.95	2.80	3.617 (2)	144

Symmetry codes: (i) 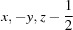; (ii) 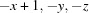; (iii) 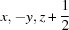.

**Fig. 1.**
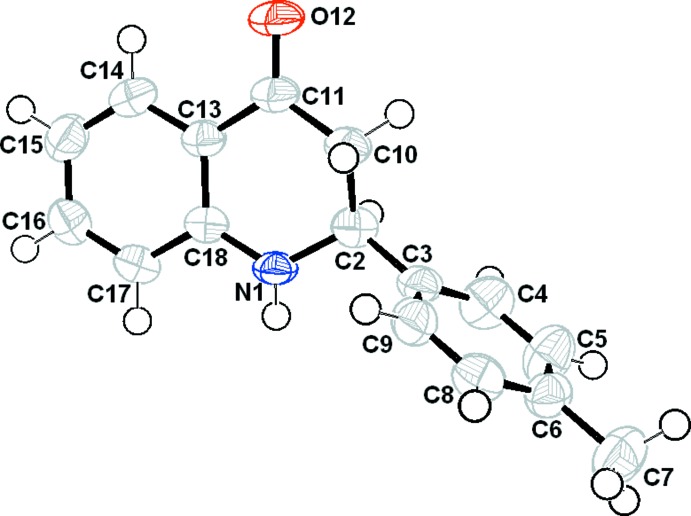
The molecular structure of (I) with displacement ellipsoids drawn at the 50% probability level. H atoms are represented as small spheres of arbitrary radius.

**Fig. 2.**
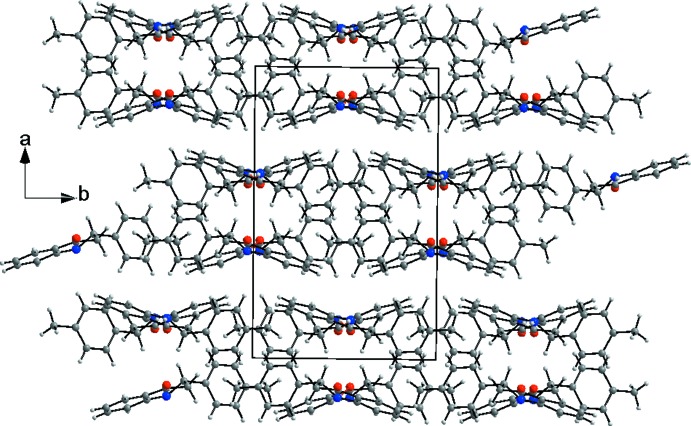
The crystal packing showing alternating double layers parallel to (100) viewed along the c axis.

**Fig. 3.**
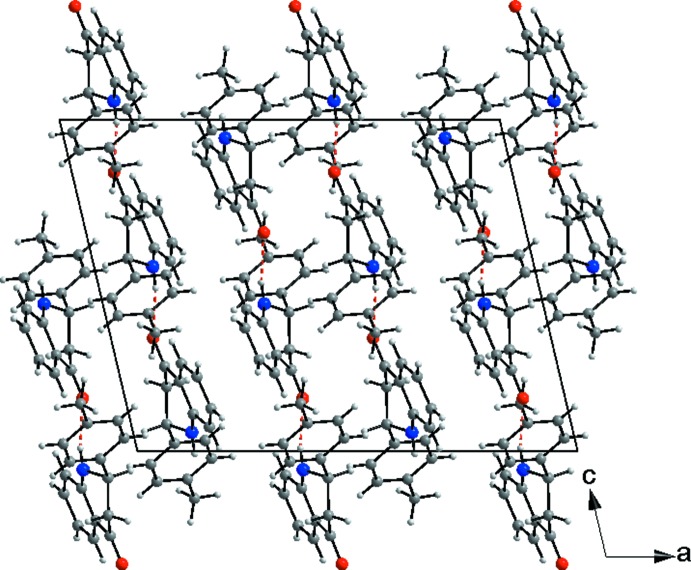
Part of the crystal structure viewed along the b axis showing hydrogen bond as dashed lines.

## supplementary crystallographic information

### 1. Comment

The role of heterocyclic compounds has become increasingly important in designing new classes of structural entities of medicinal importance. Quinolines are interesting synthetic targets because they act as building blocks for a large number of natural products (Hepworth, 1984). The formation of 2,3-dihydroquinolin-4(1*H*)-ones is generally accomplished by acid- or base-catalyzed isomerization of substituted 2'-aminochalcones (Donnelly & Farrell, 1990*a*,*b*; Tokes & Litkei, 1993). Most of these procedures involve the use of corrosive reagents such as orthophosphoric acid, acetic acid, or strong alkali. Many attempts have therefore been made to explore efficient catalysts to accelerate this reaction. Some of them are of limited synthetic scope due to low yields, long reaction times, and the need for a large amount of catalyst, specialized solvents (Tokes & Szilagyi, 1987; Tokes *et al.*, 1992), or microwave activation (Kumar *et al.*, 2004; Gordon, 2001; Olivier-Bourbigou & Magna, 2002). As part of our continuingeffort toward the development of new methods for the synthesis of biologically relevant heterocyclic compounds (Benzerka *et al.*, 2012, 2013; Hayour *et al.*, 2011), we have, recently, developed a procedure using butylmethylimidazolium(bmim).BF4 as a green solvent to provide an efficient and convenient protocol for the synthesis of 2,3-dihydroquinolin-4(1*H*)-ones from 2'-aminochalcones without the requirement for an additional catalyst (Chelghoum *et al.*, 2012). We wish to describe herein the synthesis and single-crystal X-ray structure of 2-*p*-tolyl-2,3-dihydroquinolin-4(1*H*)-one (I). The molecular structure and the atom-numbering scheme of (I) are shown in Fig. 1. The molecule of consists of a dihydroquinolin-4(1*H*)-one moiety attached to tolyl group. The (1H)-dihydropyridine ring (C2/C10/C11/C13/C18/N1) is in a sofa conformation with atom C2 forming the flap. The dihedral angle between the two benzene rings (C3-C9/C13-C18) is 80.85 (8)°. The crystal packing can be described as alternating double layers parallel to (100) (Fig. 2). Intermolecular N—H···O hydrogen bonds (Fig.3; Table. 1) link the molecules along [100]. In addition, weak C—H···π interactions are observed.

### 2. Experimental

The substituted 2'-aminochalcone (0.5 mmol) and [bmim]BF4 (1 g) were heated at 423K for 2.5 h. Under these conditions, the title compound was successfully synthesized in good yield (92%). Single crystals suitable for the X-ray diffraction analysis were obtained by dissolving the pure compound in an Et_2_O/CHCl_3_ mixture and allowing the solution to slowly evaporate at room temperature.

### 3. Refinement

All non-H atoms were refined with anisotropic atomic displacement parameters. Approximate positions for all the H atoms were first obtained from the difference electron density map. However, the H atoms were situated in idealized positions and the H-atoms were refined in a riding-motion approximation. The applied constraints were as follow: C_aryl_—H_aryl_ = 0.95 Å; C_methylene_—H_methylene_ = 0.99 Å; C_methyl_—H_methyl_ = 0.98 Å; and C_methine_—H_methine_ = 1.0 Å; The idealized methyl group was allowed to rotate about the C—C bond during the refinement by application of the command AFIX 137 in *SHELXL97* (Sheldrick, 2008). *U*_iso_(H_methyl_) = 1.5*U*_eq_(C_methyl_) or *U*_iso_(H_aryl_, _methylene_, _methine_) = 1.2 *U*_eq_(C_aryl_, _methylene_ and _methine_). Atom H1N was found in a difference electron density map and refined isotropically with *U*_iso_(H) = 1.2*U*_eq_(N)

### Crystal data

**Table:**

C_16_H_15_NO	*F*(000) = 1008
*M**_r_* = 237.29	*D*_x_ = 1.248 Mg m^−^^3^
Monoclinic, *C*2/*c*	Mo *K*α radiation, λ = 0.71073 Å
Hall symbol: -C 2yc	Cell parameters from 2437 reflections
*a* = 17.6363 (14) Å	θ = 2.4–27.5°
*b* = 10.7968 (9) Å	µ = 0.08 mm^−^^1^
*c* = 13.6308 (9) Å	*T* = 150 K
β = 103.260 (3)°	Prism, colourless
*V* = 2526.3 (3) Å^3^	0.52 × 0.33 × 0.27 mm
*Z* = 8	

### Data collection

**Table:**

Bruker APEXII diffractometer	2279 reflections with *I* > 2σ(*I*)
Graphite monochromator	*R*_int_ = 0.026
CCD rotation images, thin slices scans	θ_max_ = 27.5°, θ_min_ = 2.6°
Absorption correction: multi-scan (*SADABS*; Sheldrick, 2002)	*h* = −22→22
*T*_min_ = 0.873, *T*_max_ = 0.979	*k* = −14→10
6746 measured reflections	*l* = −17→10
2875 independent reflections	

### Refinement

**Table:**

Refinement on *F*^2^	Primary atom site location: structure-invariant direct methods
Least-squares matrix: full	Secondary atom site location: difference Fourier map
*R*[*F*^2^ > 2σ(*F*^2^)] = 0.053	Hydrogen site location: inferred from neighbouring sites
*wR*(*F*^2^) = 0.153	H atoms treated by a mixture of independent and constrained refinement
*S* = 1.05	*w* = 1/[σ^2^(*F*_o_^2^) + (0.0758*P*)^2^ + 2.2919*P*] where *P* = (*F*_o_^2^ + 2*F*_c_^2^)/3
2875 reflections	(Δ/σ)_max_ = 0.001
167 parameters	Δρ_max_ = 0.69 e Å^−^^3^
0 restraints	Δρ_min_ = −0.28 e Å^−^^3^

### Special details

**Table:**

Geometry. All e.s.d.'s (except the e.s.d. in the dihedral angle between two l.s. planes) are estimated using the full covariance matrix. The cell e.s.d.'s are taken into account individually in the estimation of e.s.d.'s in distances, angles and torsion angles; correlations between e.s.d.'s in cell parameters are only used when they are defined by crystal symmetry. An approximate (isotropic) treatment of cell e.s.d.'s is used for estimating e.s.d.'s involving l.s. planes.
Refinement. Refinement of *F*^2^ against ALL reflections. The weighted *R*-factor *wR* and goodness of fit *S* are based on *F*^2^, conventional *R*-factors *R* are based on *F*, with *F* set to zero for negative *F*^2^. The threshold expression of *F*^2^ > σ(*F*^2^) is used only for calculating *R*-factors(gt) *etc*. and is not relevant to the choice of reflections for refinement. *R*-factors based on *F*^2^ are statistically about twice as large as those based on *F*, and *R*- factors based on ALL data will be even larger.

### Fractional atomic coordinates and isotropic or equivalent isotropic displacement parameters (Å^2^)

**Table:**

	*x*	*y*	*z*	*U*_iso_*/*U*_eq_	
C2	0.58114 (9)	0.12806 (16)	0.08077 (12)	0.0263 (4)	
H2	0.5264	0.0972	0.0596	0.032*	
C3	0.58821 (10)	0.24612 (15)	0.02362 (12)	0.0260 (4)	
C4	0.52341 (10)	0.29679 (18)	−0.03934 (14)	0.0346 (4)	
H4	0.4744	0.2568	−0.0469	0.042*	
C5	0.52839 (11)	0.40454 (19)	−0.09163 (15)	0.0378 (4)	
H5	0.4824	0.438	−0.1337	0.045*	
C6	0.59864 (10)	0.46561 (16)	−0.08463 (12)	0.0273 (4)	
C7	0.60403 (12)	0.58261 (18)	−0.14265 (15)	0.0402 (5)	
H7A	0.6587	0.608	−0.1317	0.06*	
H7B	0.574	0.6483	−0.1194	0.06*	
H7C	0.5829	0.5677	−0.2147	0.06*	
C8	0.66448 (9)	0.41410 (17)	−0.02209 (12)	0.0281 (4)	
H8	0.7136	0.4532	−0.0161	0.034*	
C9	0.65959 (10)	0.30599 (17)	0.03197 (12)	0.0295 (4)	
H9	0.7052	0.2727	0.0749	0.035*	
C10	0.60048 (10)	0.14901 (16)	0.19407 (11)	0.0284 (4)	
H10A	0.6503	0.1948	0.2132	0.034*	
H10B	0.5595	0.2018	0.2113	0.034*	
C11	0.60719 (10)	0.03170 (16)	0.25526 (12)	0.0269 (4)	
C13	0.63292 (9)	−0.07957 (16)	0.21014 (11)	0.0233 (3)	
C14	0.64681 (10)	−0.19096 (17)	0.26412 (12)	0.0296 (4)	
H14	0.6365	−0.1956	0.3294	0.035*	
C15	0.67503 (10)	−0.29353 (17)	0.22452 (13)	0.0312 (4)	
H15	0.6843	−0.3684	0.262	0.037*	
C16	0.68997 (9)	−0.28610 (16)	0.12829 (13)	0.0287 (4)	
H16	0.7099	−0.3564	0.1007	0.034*	
C17	0.67624 (9)	−0.17860 (16)	0.07284 (12)	0.0253 (4)	
H17	0.6868	−0.1755	0.0076	0.03*	
C18	0.64660 (8)	−0.07303 (15)	0.11196 (11)	0.0206 (3)	
N1	0.63337 (8)	0.03393 (13)	0.05626 (10)	0.0229 (3)	
H1N	0.6284 (11)	0.0219 (18)	−0.0085 (16)	0.028*	
O12	0.59607 (9)	0.03372 (13)	0.34096 (9)	0.0416 (4)	

### Atomic displacement parameters (Å^2^)

**Table:**

	*U*^11^	*U*^22^	*U*^33^	*U*^12^	*U*^13^	*U*^23^
C2	0.0283 (8)	0.0280 (9)	0.0236 (8)	−0.0016 (7)	0.0076 (6)	−0.0007 (6)
C3	0.0363 (9)	0.0234 (8)	0.0213 (7)	0.0002 (7)	0.0130 (6)	−0.0022 (6)
C4	0.0292 (8)	0.0348 (10)	0.0393 (10)	−0.0087 (7)	0.0068 (7)	0.0020 (8)
C5	0.0291 (9)	0.0382 (11)	0.0413 (10)	−0.0014 (8)	−0.0018 (7)	0.0088 (8)
C6	0.0341 (9)	0.0227 (8)	0.0257 (8)	−0.0020 (7)	0.0080 (6)	−0.0003 (6)
C7	0.0520 (11)	0.0280 (10)	0.0395 (10)	−0.0034 (8)	0.0084 (8)	0.0063 (8)
C8	0.0237 (7)	0.0313 (9)	0.0301 (8)	−0.0046 (7)	0.0075 (6)	−0.0040 (7)
C9	0.0291 (8)	0.0331 (10)	0.0252 (8)	0.0069 (7)	0.0042 (6)	0.0009 (7)
C10	0.0406 (9)	0.0267 (9)	0.0198 (7)	0.0029 (7)	0.0110 (7)	−0.0022 (6)
C11	0.0333 (8)	0.0314 (9)	0.0166 (7)	0.0006 (7)	0.0072 (6)	−0.0006 (6)
C13	0.0255 (7)	0.0265 (8)	0.0179 (7)	−0.0004 (6)	0.0054 (6)	−0.0005 (6)
C14	0.0353 (9)	0.0329 (10)	0.0210 (7)	0.0009 (7)	0.0074 (6)	0.0050 (7)
C15	0.0338 (9)	0.0278 (9)	0.0315 (9)	0.0023 (7)	0.0063 (7)	0.0063 (7)
C16	0.0277 (8)	0.0260 (9)	0.0339 (9)	0.0014 (7)	0.0098 (7)	−0.0027 (7)
C17	0.0265 (8)	0.0277 (9)	0.0238 (7)	−0.0021 (6)	0.0100 (6)	−0.0035 (6)
C18	0.0197 (7)	0.0236 (8)	0.0187 (7)	−0.0041 (6)	0.0047 (5)	−0.0022 (6)
N1	0.0313 (7)	0.0236 (7)	0.0159 (6)	−0.0003 (5)	0.0097 (5)	−0.0013 (5)
O12	0.0695 (10)	0.0406 (8)	0.0189 (6)	0.0102 (7)	0.0186 (6)	0.0020 (5)

### Geometric parameters (Å, º)

**Table:**

C2—N1	1.461 (2)	C10—C11	1.506 (2)
C2—C3	1.514 (2)	C10—H10A	0.99
C2—C10	1.520 (2)	C10—H10B	0.99
C2—H2	1	C11—O12	1.228 (2)
C3—C4	1.376 (2)	C11—C13	1.468 (2)
C3—C9	1.396 (2)	C13—C14	1.402 (2)
C4—C5	1.378 (3)	C13—C18	1.415 (2)
C4—H4	0.95	C14—C15	1.374 (3)
C5—C6	1.387 (2)	C14—H14	0.95
C5—H5	0.95	C15—C16	1.398 (2)
C6—C8	1.389 (2)	C15—H15	0.95
C6—C7	1.505 (2)	C16—C17	1.376 (2)
C7—H7A	0.98	C16—H16	0.95
C7—H7B	0.98	C17—C18	1.409 (2)
C7—H7C	0.98	C17—H17	0.95
C8—C9	1.394 (2)	C18—N1	1.372 (2)
C8—H8	0.95	N1—H1N	0.88 (2)
C9—H9	0.95		
			
N1—C2—C3	109.72 (13)	C11—C10—C2	114.09 (14)
N1—C2—C10	109.24 (13)	C11—C10—H10A	108.7
C3—C2—C10	111.82 (14)	C2—C10—H10A	108.7
N1—C2—H2	108.7	C11—C10—H10B	108.7
C3—C2—H2	108.7	C2—C10—H10B	108.7
C10—C2—H2	108.7	H10A—C10—H10B	107.6
C4—C3—C9	118.11 (15)	O12—C11—C13	123.04 (15)
C4—C3—C2	120.08 (15)	O12—C11—C10	120.15 (15)
C9—C3—C2	121.81 (15)	C13—C11—C10	116.66 (13)
C3—C4—C5	121.08 (16)	C14—C13—C18	119.47 (15)
C3—C4—H4	119.5	C14—C13—C11	121.05 (14)
C5—C4—H4	119.5	C18—C13—C11	119.45 (14)
C4—C5—C6	121.84 (16)	C15—C14—C13	121.38 (15)
C4—C5—H5	119.1	C15—C14—H14	119.3
C6—C5—H5	119.1	C13—C14—H14	119.3
C5—C6—C8	117.36 (16)	C14—C15—C16	119.03 (16)
C5—C6—C7	121.72 (16)	C14—C15—H15	120.5
C8—C6—C7	120.92 (16)	C16—C15—H15	120.5
C6—C7—H7A	109.5	C17—C16—C15	121.09 (16)
C6—C7—H7B	109.5	C17—C16—H16	119.5
H7A—C7—H7B	109.5	C15—C16—H16	119.5
C6—C7—H7C	109.5	C16—C17—C18	120.56 (14)
H7A—C7—H7C	109.5	C16—C17—H17	119.7
H7B—C7—H7C	109.5	C18—C17—H17	119.7
C6—C8—C9	121.02 (15)	N1—C18—C17	120.15 (13)
C6—C8—H8	119.5	N1—C18—C13	121.39 (14)
C9—C8—H8	119.5	C17—C18—C13	118.44 (14)
C8—C9—C3	120.57 (15)	C18—N1—C2	119.67 (12)
C8—C9—H9	119.7	C18—N1—H1N	113.4 (13)
C3—C9—H9	119.7	C2—N1—H1N	114.2 (13)
			
N1—C2—C3—C4	122.88 (17)	C10—C11—C13—C14	175.01 (15)
C10—C2—C3—C4	−115.76 (17)	O12—C11—C13—C18	−178.14 (16)
N1—C2—C3—C9	−56.54 (19)	C10—C11—C13—C18	−2.7 (2)
C10—C2—C3—C9	64.8 (2)	C18—C13—C14—C15	1.4 (2)
C9—C3—C4—C5	−0.9 (3)	C11—C13—C14—C15	−176.33 (15)
C2—C3—C4—C5	179.62 (17)	C13—C14—C15—C16	−0.1 (3)
C3—C4—C5—C6	1.0 (3)	C14—C15—C16—C17	−0.6 (3)
C4—C5—C6—C8	−0.3 (3)	C15—C16—C17—C18	0.0 (2)
C4—C5—C6—C7	179.37 (18)	C16—C17—C18—N1	179.75 (14)
C5—C6—C8—C9	−0.6 (3)	C16—C17—C18—C13	1.3 (2)
C7—C6—C8—C9	179.79 (16)	C14—C13—C18—N1	179.63 (14)
C6—C8—C9—C3	0.7 (3)	C11—C13—C18—N1	−2.6 (2)
C4—C3—C9—C8	0.1 (2)	C14—C13—C18—C17	−1.9 (2)
C2—C3—C9—C8	179.54 (15)	C11—C13—C18—C17	175.80 (14)
N1—C2—C10—C11	−48.91 (19)	C17—C18—N1—C2	160.66 (14)
C3—C2—C10—C11	−170.55 (14)	C13—C18—N1—C2	−20.9 (2)
C2—C10—C11—O12	−155.13 (16)	C3—C2—N1—C18	168.89 (13)
C2—C10—C11—C13	29.3 (2)	C10—C2—N1—C18	45.99 (19)
O12—C11—C13—C14	−0.4 (3)		

### Hydrogen-bond geometry (Å, º)

Cg1 and Cg2 are the centroids of the C13–C18 and C3–C9 rings, respectively.

**Table:**

*D*—H···*A*	*D*—H	H···*A*	*D*···*A*	*D*—H···*A*
N1—H1*N*···O12^i^	0.88 (2)	2.09 (2)	2.9484 (17)	166.9 (18)
C4—H4···*Cg*1^ii^	0.95	2.70	3.546 (2)	149
C14—H14···*Cg*2^iii^	0.95	2.80	3.617 (2)	144

Symmetry codes: (i) *x*, −*y*, *z*−1/2; (ii) −*x*+1, −*y*, −*z*; (iii) *x*, −*y*, *z*+1/2.
